# Germline mutation of TSC1 or TSC2 gene in Chinese patients with bilateral renal angiomyolipomas and mutation spectrum of Chinese TSC patients

**DOI:** 10.18632/aging.102654

**Published:** 2020-01-12

**Authors:** Wang Jiangyi, Guo Gang, Shi Guohai, Ye Dingwei

**Affiliations:** 1 Department of Urology, Fudan University Shanghai Cancer Center, Shanghai, PR China; 2 Department of Oncology, Shanghai Medical College, Fudan University, Shanghai, PR China; 3 Department of Urology, the State Key Laboratory of Kidney Diseases, Chinese PLA General Hospital, Beijing, PR China

**Keywords:** angiomyolipoma, tuberous sclerosis complex, mutation spectrum, gene test

## Abstract

The germline mutation of the TSC1/2 gene in bilateral renal angiomyolipomas is unclear. Meanwhile, the mutation spectrum of Chinese TSC patients has not been revealed. We recruited 78 patients diagnosed with bilateral renal AMLs. High-throughput sequencing was used to detect any variants in TSC1/2 genes. The results showed that 28.6% of patients diagnosed before 45 were with positive results of TSC1/2 test. The rate decreased to 14.3% for those with onset age over 45. For the 315 previously reported Chinese patients, TSC1 patients were more likely to be affected by nonsense mutations (51.1% vs. 20.7%, p<0.001) and had a significantly higher rate of family history than TSC2 patients (37.8% vs. 19.6%, p=0.0067). Moreover, exon8, 15, and 18 were the hotspot mutation regions for TSC1, and exon 29, 33 and 40 were the most common mutation regions for TSC2. Besides, Chinese TSC patients carried more TSC2 alterations (85.7% vs.76.2%, p<0.001), and were more likely to have a family history than those from TOSCA (22.2% vs. 13.9%, p<0.001). In conclusion, patients affected by bilateral renal AMLs should receive genetic testing of TSC ½ genes and Chinese TSC patients have relatively hotspot mutation regions, which are helpful to genetic counseling and clinical decision making.

## INTRODUCTION

Angiomyolipoma (AML) is one of the most common benign solid tumors in the kidney [1]. Histologically, it is characterized by different proportions of blood vessels, smooth muscle, and fat [2]. However, the epithelioid AML, especially the pure epithelioid renal AML (PECOMA), could be potentially aggressive [3]. AML tends to be more common in female patients with a female to male ratio of 2:1 [4]. Abdominal pain and hematuria are the most reported symptoms. With the widespread use of CT and ultrasound, more incidental AMLs are diagnosed [5].

About 80% of renal AMLs are sporadic, and the rest are associated with hereditary tumor syndrome, such as tuberous sclerosis complex (TSC) [2]. TSC is an autosomal dominant tumor syndrome caused by the germline mutation of TSC1/2, with a birth incidence of 1 in 10000 to 1 in 6000 [6]. Patients are predisposed to types of tumors in different organs in their early ages, including the kidney, brain, skin, and lung [7]. Renal AML (RAMLs) is found in over half of TSC patients and is one of the major criteria for the clinical diagnostic criterion recommended by the International Consensus Conference in 2012 [8, 9]. TSC-related RAMLs show different phenotype from the sporadic ones, and are characterized by the early onset age (20–30 years old), bilateral and multifocal lesions, and concurrent tumors in skin, brain and lung [10]. Moreover, the TSC-related RAMLs are reported to be with a larger initial size, grow faster and have higher a risk of spontaneous hemorrhage, which can be life threatening, than the sporadic ones [11]. Thus, it is important to recognize the patients with TSC from those affected by RAMLs.

Since the clinical diagnostic criterion can only be used after the TSC-related tumors occur, clinicians now try to use the next generation sequencing (NGS) techniques to identify TSC patients at an early stage. A positive pathogenic TSC1/2 germline mutation will lead to a definite diagnosis of TSC [8]. According to the 2012 International TSC Consensus, patients with bilateral or multifocal RAMLs are recommended to take genetic testing. However, no evidence has been reported about the germline TSC1/2 gene status among patients with bilateral RAMLs. Besides, the NGS strategy is unreliable for the detection of the large deletion/amplification, which needs confirmation of the multiplex ligation-dependent probe amplification (MLPA). The standard genetic testing method is not suggested in the existing guidelines and should be chosen according to the mutation spectrum of one region. Unfortunately, The Chinese TSC1/2 mutation spectrum is still uncertain.

In this study, we evaluated the status of TSC1/2 genes among patients with bilateral RAMLs and found that about ¼ of patients carried a germline TSC1/2 variant. The result, for the first time, provided evidence for these patients to take genetic testing. Meanwhile, we described the mutation spectrum of TSC1/2 genes among Chinese TSC patients and found that almost all types of TSC1/2 variants occurred in Chinese patients. Thus NGS based techniques combined with MLPA should be recommended to be the standard genetic testing strategy for Chinese patients with TSC. These results will be helpful for the clinical-decision-making of bilateral renal AMLs and provide valuable information for genetic counseling for those Chinese patients with TSC.

## RESULTS

### Clinical characteristics of patients with bilateral RAML in this study

A total of 78 individuals were enrolled for analysis, including 28 male and 50 female patients. The baseline information was demonstrated in Table 1. The onset age of RAML in our study was 32.5±14.5 years old (median 29.5, range 1 to 77), which was much earlier than the sporadic RAMLs [4]. Among all the patients, 62 patients were diagnosed before 45 years, while the other 16 ones were affected over 45. Most of the patients (73/78) had no family history. A total of 49 patients were affected by only bilateral RAMLs, while the other 29 patients were affected by bilateral RAMLs and at least one TSC-related tumors (Table 1).

**Table 1:** Clinical and genetic characteristics of patients with bilateral renal AMLs.

**Characteristics**	**No. of patients**
Sex	
male	28
female	50
Onset age of AML	
≤45y	62
>45y	16
Family history	
yes	5
no	73
Concomitant tumors	
yes	29
no	49
TSC1/2 gene test	
positive	30
negative	48

### Germline mutation of TSC1 or TSC2 gene in different groups of bilateral RAML patients

We carried out next-generation sequencing to detect the status of TSC1/2 genes and found 30 germline alterations (3 TSC1 and 27 TSC2). For early-onset patients who were diagnosed before 45 years old, 28.6% of them were with positive results of the TSC1/2 test. The rate decreased to 14.3% (2 out of 14) for those with onset age over 45 years old. As to the 29 patients affected by both bilateral RAMLs and other TSC-related tumors, which met the clinical criteria for TSC, 18 (62.1%) alterations in TSC1/2 were observed (Table 2).

**Table 2:** Germline mutation of TSC1/2 in different groups of patients with bilateral RAMLs.

**Clinical features**	**TSC1/2 gene test**		**Total**
	**+**	**-**	
Bilateral RAML			
≤45y	10(28.6%)	25 (71.4%)	35
>45y	2 (14.3%)	12 (85.7%)	14
Total	12 (24.5%)	37 (75.5%)	49
Bilateral RAML with other TSC-related tumors	18(62.1%)	11 (37.9%)	29
Total	30	48	78

Among the 30 alterations, 24 were pathogenic or likely pathogenic according to the ACMG guideline, and the others were variants of uncertain significance (VUS). To illustrate the effect of the 6 VUS on protein function, we carried out the *in silico* prediction programs and found that 5/6 of the VUS were predicted to be harmful. The last one VUS was a synonymous mutation predicted to be benign, but according to the clinical criteria, that patient was diagnosed with TSC. We supposed this mutation might lead to TSC syndrome with a special pathogenetic mechanism. Moreover, most of the alterations were truncating, including eight frameshift mutations, eight nonsense mutations, four splicing mutations, and two deletion/duplications. The mutations in the TSC1 or TSC2 gene were compared with those in the Tuberous Sclerosis Database (http://chromium.lovd.nl/LOVD2/TSC/home), and 15 (50%) of them were first reported in this study. The detailed genotypic information and clinical manifestations were shown in Table 3.

**Table 3:** Genotypic and phenotypic features of patients with germline mutation of TSC1/2.

**No.**	**Gene**	**Nucleotide change**	**Protein change**	**Mutation type**	**Novel**	**Clinical manifestation**	**ACMG**
1	TSC2/E5	c.433G>T	p. Arg611Trp	Missense	Novel	RAML, ungual fibromas	Likely pathogenic
2	TSC2/E11	EX11 DEL	—	Deletion	Novel	RAML, angiofibroma, forehead plaque, shagreen patch, SEGA, seizures	Likely pathogenic
3	TSC2/E11_15	E11-15 DUP	—	Deletion	Novel	RAML, angiofibroma,	Likely pathogenic
4	TSC2/E14	c.1372C>T	p. Arg458Ter	Nonsense	Reported	RAML	Likely pathogenic
5	TSC2/E15	c.1513C>T	p. Arg505Ter	Nonsense	Reported	RAML, angiofibroma	Likely pathogenic
6	TSC2/E17	c.1831C>T	p. Arg611Trp	Missense	Reported	RAML, angiofibroma, ungual fibromas	Likely pathogenic
7	TSC2/E19	c.2083C>T	p. Gln695Ter	Nonsense	Reported	RAML	Likely pathogenic
8	TSC2/E20	c.2102_2105 delCTGA	p. Ser701Ser fsX5	Frameshift	Reported	RAML	Likely pathogenic
9	TSC2/E20	c.2138T>C	p. Leu713Pro	Missense	Novel	RAML, angiofibroma	VUS
10	TSC2/E27	c.3046delA	p. Lys1061Lys fs X14	Frameshift	Novel	RAML, seizures	Likely pathogenic
11	TSC2/E30	c.3412C>T	p. Arg1138Ter	Nonsense	Reported	RAML	Likely pathogenic
12	TSC2/E30	c.3412C>T	p. Arg1138Ter	Nonsense	Reported	RAML, seizures	Likely pathogenic
13	TSC2/E30	c.3582G>A	p. Trp1194Ter	Nonsense	Reported	RAML	Likely pathogenic
14	TSC2/E31	c.3685C>T	p. Gln1229Ter	Nonsense	Reported	RAML, angiofibroma, ungual fibromas, Hypomelanotic macules, seizures	Likely pathogenic
15	TSC2/E31	c.3803G>A	p. Arg1268His	Missense	Novel	RAML, angiofibroma	VUS
16	TSC2/E34	c.4418_4419 delAG	p. Lys1473Lys fsX50	Frameshift	Reported	RAML, angiofibroma	Likely pathogenic
17	TSC2/E34	c.4425_4426 delAG	p. Arg1477Gly fs X46	Frameshift	Reported	RAML, angiofibroma	Likely pathogenic
18	TSC2/E34	c.4493_c.4493+18 delGGTGGGCCTCTTGCTTCCG	—	Frameshift	Novel	RAML, forehead plaque,	Likely pathogenic
19	TSC2/E37	c.4737C>T	p. Gly1579Gly	Missense	Novel	RAML, angiofibroma,	VUS
20	TSC2/E37	c.4783G>A	p. Gly1595Arg	Missense	Novel	RAML, angiofibroma	VUS
21	TSC2/E40	c.5155G>C	p. Ala1719Pro	Missense	Novel	RAML, angiofibroma	VUS
22	TSC2/E41	c.5175_5176del/GC	p. His1726Ser fsX2	Frameshift	Novel	RAML, angiofibroma	Likely pathogenic
23	TSC2/E41	c.5237_5238insC	p. His1746His fsX29	Frameshift	Novel	RAML, angiofibroma, angual fibromas, Hypomelanotic macules, shagreen patch, seizures	Likely pathogenic
24	TSC2/IN9	c.849-1G>A	—	Splicing	Reported	RAML	Likely pathogenic
25	TSC2/IN14	c.1444-1G>C	—	Splicing	Novel	RAML, forehead plaque,	Likely pathogenic
26	TSC2/IN15	c.1600-1G>C	—	Splicing	Novel	RAML, angiofibroma,	Likely pathogenic
27	TSC2/IN30	c.3610+1G>A	—	Splicing	Reported	RAML	Likely pathogenic
28	TSC1/E6	c.372delT	p. Thr124Thr fsX13	Frameshift	Novel	RAML	Likely pathogenic
29	TSC1/E6	c.433C>T	p. Gln145Ter	Nonsense	Reported	RAML	Pathogenic
30	TSC1/E15	c.1960C>G	p. Gln654Glu	Missense	Reported	RAML	VUS

### TSC1/2 mutation spectrum in Chinese patients and the genotypic and phenotypic characteristics

**Figure 1:**
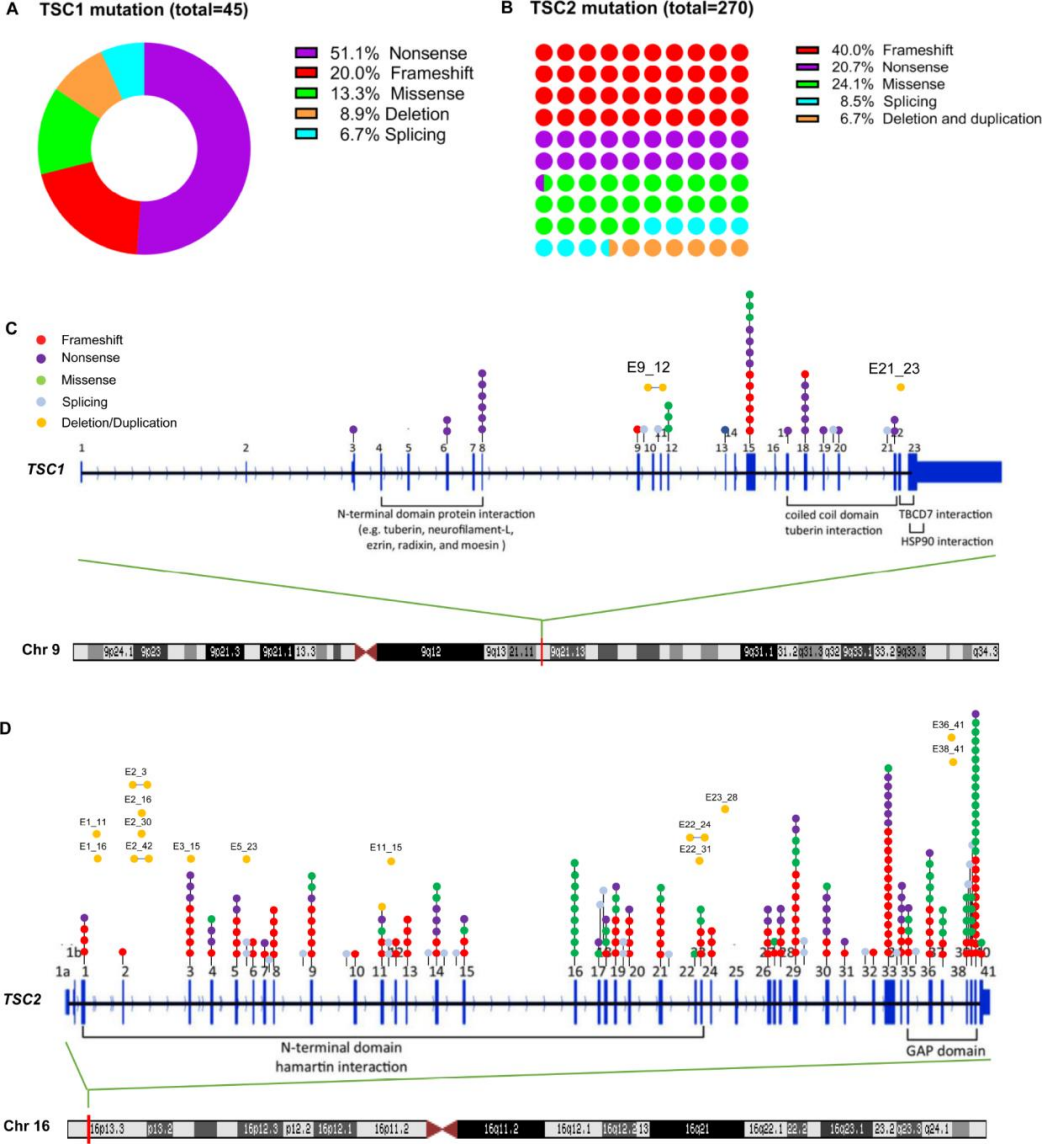
**TSC1 and TSC2 gene mutation spectrum in Chinese patients.** (**A**) mutation types of TSC1 gene; (**B**) mutation types of TSC2 gene; (**C**) mutation sites of TSC1 gene; (**D**) mutation sites of TSC2 gene.

To demonstrate the mutation spectrum of Chinese TSC patients, we reviewed all the reported TSC patients in China. A total of 315 alterations were involved for analysis, including 45 TSC1 and 270 TSC2 (Supplementary Table 1). TSC1 patients were more likely to be affected by nonsense mutations than TSC2 patients (51.1% vs. 20.7%, p<0.001), while the frameshift and missense mutations were more common in TSC2 patients (40% vs. 20%, 24.1% vs. 13.3%) (Figure 1A and 1B). Moreover, patients with TSC1 mutation had a significantly higher positive rate of family history compared to those with TSC2 mutation (37.8% vs. 19.6%, p=0.0067) (Table 4). The mutation spectrum of patients with TSC1 or TSC2 gene was next analyzed. For the TSC1 gene, exon 15, 8 and 18 seemed to be the hotspot mutation regions. For the TSC2 gene, we observed alterations in every exon except for exon 25. Specifically, exon 40, 33, and 29 were the most common mutation regions, which accounted for about 30% (60) of all the variants (Figure 1C and 1D).

**Table 4:** Differences of mutation types and family history between Chinese patients carrying TSC1 and TSC2 mutations.

	**TSC1**	**TSC2**	**P value**
**Mutation type**			
Frameshift	9 (20%)	108 (40%)	0.0004
Nonsense	23 (51.1%)	56 (20.7%)	
Missense	6 (13.3%)	65 (24.1%)	
Splicing	3 (6.7%)	23 (8.5%)	
Deletion and duplication	4 (8.9%)	18 (6.7%)	
Total	45	270
**Family history**			
Positive	17 (37.8)	53 (19.6%)	0.0067
Negative	28 (62.2%)	217 (80.4%)
Total	45	270

To clarify the genotypic and phenotypic differences between TSC patients from China and the western countries, we analyzed the data of sex, family history, and mutated genes in Chinses TSC patients and the TOSCA cohort. The results showed that 85.7% of Chinese TSC patients carried TSC2 alterations, which was higher than that of TOSCA (76.2%, p<0.001) (Table 5). Moreover, Chinese TSC patients were more likely to have an affected parent than those from TOSCA (22.2% vs. 13.9%, p<0.001) (Table 5). Female TSC patients were more common than male patients, but no difference was observed between the Chinese cohort and TOSCA cohort.

**Table 5:** Differences in mutation spectrum and phenotypic characteristics between TSC patients in China and the TOSCA project.

	**China**	**TOSCA**	**P value**
**Gene mutation**			
TSC1	45 (14.3%)	178 (23.8%)	0.0005
TSC2	270(85.7%)	571 (76.2%)	
**Family history**			
positive	70 (22.2%)	290 (13.9%)	0.0001
negative	245 (77.8%)	1803 (86.1%)	
**Sex**			
male	88 (47.6%)	1009 (48.2%)	0.867
female	97 (52.4%)	1084 (51.8%)	

## DISCUSSION

Renal AML is a relatively rare tumor with an overall prevalence of 0.1% to 0.44%. Only 5.2% of the patients present with multiple AMLs, and about 1.5% of them are bilateral [4]. According to the current guidelines, active surveillance is the most chosen option for most of the sporadic renal AMLs for the low growth rate and low probability of spontaneous rupture of the tumors, no matter what the initial tumor size is. And nephron-sparing surgery is the most effective treatment to reduce the rate of recurrence and secondary treatment [12]. However, for the TSC associated RAMLs, mTOR inhibitor treatment is the recommended first-line therapy for asymptomatic tumors larger than 3 cm [13, 14]. Thus, the differentiation of the TSC associated RAML and the sporadic one is of great importance for patients. Nowadays, patients with unilateral RAML are not recommended to take genetic testing if they do not develop other TSC-related tumors at the same time. But, when bilateral or multifocal RAMLs exist, gene testing is recommended according to the 2012 International TSC Consensus [8, 14]. However, the positive rate of genetic testing for patients with bilateral RAMLs remained unclear. In our study, we observed that ¼ patients with bilateral RAMLs carried TSC1/2 germline alterations. Moreover, the positive probability increased to 28% when patients were affected before 45 years old. Meanwhile, in our previous study, about 10% of patients with early-onset (<45 years old) unilateral AML were found to carry TSC1/2 germline alterations [15]. These results provide strong evidence for the utility of TSC1/2 testing for all patients with bilateral RAMLs and those with early-onset unilateral RAML.

Genotype-phenotype correlations in TSC syndrome have been studied well in the past decades. Generally, TSC2 pathogenic variants are related to a more severe phenotype than TSC1 [16]. As to renal lesions, patients carrying TSC2 mutations usually have larger initial tumor size, higher risk for a growing RAML, and higher risk for hemorrhage [17]. Meanwhile, the onset age of RAML among patients with TSC2 germline mutations are almost ten years earlier than those with TSC1 mutations [18]. Although the genotype-phenotype correlations are still challenging because of the age dependence of onset of TSC-associated manifestations, they provide valuable information for genetic counseling and give us a clue for the pathogenesis of TSC-related tumors. In this study, we observed that only a small proportion of TSC patients inherited the mutated gene from their parent, and positive family history was more common among patients with TSC1 germline mutations compared to those with TSC2 mutations. This phenotypic diversity can be partly explained by the poorer prognosis of patients carrying TSC2 mutations. In most circumstances, the severe symptoms and early onset age make the patients unable to bear a child or unwilling to give birth to an unhealthy baby when the prenatal diagnosis or preimplantation genetic diagnosis is not available. The difference in genetic anticipation between TSC1 and TSC2 families may also play a role in the phenotypic disparity. Future studies on the genetic and clinical characteristics of successive generations among TSC1 and TSC2 families are needed to confirm the hypothesis.

In line with the previous study, most of the alterations observed in our study were truncating variants, which indicates that the complete loss of function of the TSC1/2 genes is essential for the tumorigenesis of most TSC associated lesions. Interestingly, we found a silent mutation located in exon 37 of the TSC2 gene (c.4737C>T), which led to the alteration from GGC to GGT without transforming the amino acid. Silent mutations are generally considered to be normal variants and are thought to have no role in disease. However, that patient carrying this variation had been diagnosed with TSC syndrome based on the clinical criteria. In order to clarify the other atypical mutation sites in TSC1/2 genes, we screened the whole exons and the surrounding introns and found that the c.4737C>T was the only alteration of TSC1/2 genes. Although the pathogenesis of silent mutation in TSC syndrome has not been found, synonymous mutations in other genes have been reported to influence gene expression during pre-mRNA splicing and play a role in disease through the exonic splicing enhancer (ESE) and exonic splicing silencer motifs [19]. Thus, the c.4737C>T alteration may cause the skipping or retention of related exons in TSC2, leading to the dysfunction of protein. More experimental work needs to be done the reveal the detailed mechanism.

In the past several years, the NGS based methods have been recommended to screen germline or somatic alterations of TSC1/2 genes in selected individuals. However, MLPA is usually recommended in the condition of a negative result of NGS. The mutation spectrum of TSC1/2 genes in Chinese TSC patients was demonstrated in our study. Almost all kinds of mutation types were found, including frameshift mutations, nonsense mutations, small indels, large deletions/amplifications, splicing mutations, and synonymous mutations. Thus, the NGS based methods, followed by MLPA if negative, should be the routine strategies for patients suspected to be affected by TSC.

In conclusion, patients affected by merely bilateral RAMLs should receive genetic testing of TSC ½ genes, especially when they are with an early onset age. Meanwhile, the mutation spectrum of Chinese TSC patients suggests that NGS based methods and MLPA should be the recommended strategies of genetic testing. The results will be helpful to genetic counseling in clinical decision making and help to reduce the missed diagnosis rate of TSC related RAMLs and to provide accurate therapeutic strategies for patients.

## MATERIALS AND METHODS

### Medical ethics

This project was approved by the scientific and ethics committee of the Fudan University Shanghai Cancer Center. Informed consent was obtained from patients or legal guardians.

### Patient ascertainment and assessment

In this study, we recruited consecutive patients from Jan 2018 to Jan 2019, who met the following criteria: diagnosed with bilateral renal AML by image test or pathology; agreed to receive the genetic test. A total of 78 patients who met the above criteria were enrolled for analysis. Clinical characteristics including birth year, sex, onset age, and family history were collected through interviews with the probands and check of medical records. The onset age was defined as the diagnosed age of first renal AML if the patient was affected by metachronous bilateral AMLs. Positive family history was identified when one of the parents was diagnosed with TSC by the clinical criteria recommended by the International Consensus Conference in 2012, or with a pathogenic germline TSC1/2 alteration.

### DNA preparation and NGS

Genomic DNA was extracted from peripheral blood of enrolled patients according to the standard procedure using the QIAamp DNA Blood Midi Kit (Qiagen, Hilden, Germany). Then the DNA was fragmented by Covaris LE220 (Massachusetts, USA) to generate a paired-end library (200–250 bp). The library was enriched by array hybridization according to previously published procedure [20], followed by elution and post-capture amplification. We next estimated the magnitude of enrichment with Agilent 2100 Bioanalyzer and ABI StepOne. All amplified libraries were subsequently sent to BGI for circularization and sequencing on the BGISEQ-500 platform. For circularization, PCR products with different barcodes were pooled together at an equimolar concentration to yield a final amount of 80 ng. Each pool was subsequently heat-denatured, and the single-strand DNA was mixed with MGIEasyTM DNA Library Prep Kit V1 (PN:85–05533-00, BGI, Shenzhen, China), containing 5 μl splint oligo, 6 μl splint Buffer, 0.6 μl ligation Enhancer, and 0.2 μl ligation (Enzyme and NF water) to form a 60 μl reaction system, which was subsequently incubated at 37°C for 30 minutes. Last, 20 μl of each single-circle-library pool was used as input to prepare the DNB. Each pool was then sequenced on 1 lane, using 100SR chemistry with BGISEQ-500RS High-throughput sequencing kit (PN: 85–05238-01, BGI). Post sequencing, the data were automatically demultiplexed by index.

### Bioinformatics and variant filtering

To detect the potential variants in the family, we performed bioinformatics processing and data analysis after receiving the primary sequencing data. We used previously published filtering criteria to generate “clean reads” for further analysis [20]. The “clean reads” (with a length of 90 bp) derived from targeted sequencing and filtering were then aligned to the human genome reference (hg19) using the BWA (Burrows-Wheeler Aligner) Multi-Vision software package [21]. After alignment, the output files were used to perform sequencing coverage and depth analysis of the target region, single-nucleotide variants (SNVs) and INDEL calling. We used SOAPsnp software [22] and Samtools [23] to detect SNVs and indels. All SNVs and indels were filtered and estimated via multiple databases, including NCBI dbSNP, HapMap, 1000 human genome dataset, and database of 100 healthy Chinese adults.

To predict the effect of missense variants, we used dbNSFP [24], which contains eleven well-established in silico prediction programs. Pathogenic variants were assessed under the protocol issued by ACMG [25]. The Human Gene Mutation Database (HGMD) was used to screen mutations reported in published studies.

All mutations and potential pathogenic variants were validated using conventional Sanger sequencing methods. The PCR protocol consisted of an initial denaturation at 95°C for 3 minutes, followed by 35 cycles (95°C for 40 seconds, 55°C for 30 seconds, and 72°C for 30 seconds), with a final extension at 72°C for 10 minutes.

### Statistical analysis

Chi-square test was used to compare the differences in mutation type and family history between patients with TSC1 and TSC2 gene mutation, and to compare the differences of the mutated gene, family history and sex between TSC patients from China and the TOSCA cohort. Statistical analysis was performed using SPSS20.0, and p < 0.05 was considered to be statistically significant.

## Supplementary Material

Supplementary Table 1
